# Whole-genome sequence data of a multidrug-resistant *Klebsiella pneumoniae* strain KP-S-66 isolated from cattle shed soil

**DOI:** 10.1016/j.dib.2026.112967

**Published:** 2026-06-11

**Authors:** Sonia Akther, Md Abu Ahsan Gilman, Md. Niamul Shahadat, Mst. Sogra Banu Juli, M. Nazmul Hoque

**Affiliations:** a Bangladesh Livestock Research Institute, Savar, Dhaka, 1341, Bangladesh; b Molecular Biology and Bioinformatics Laboratory, Department of Gynecology, Obstetrics and Reproductive Health, Gazipur Agricultural University, Gazipur 1706, Bangladesh; c Faculty of Veterinary and Animal Sciences, Hajee Mohammad Danesh Science and Technology University, Dinajpur, 5200, Bangladesh

**Keywords:** *K. pneumoniae*, Genome sequencing, Resistome, Efflux pump, Cattle farm, Soil

## Abstract

*Klebsiella pneumoniae* is a major opportunistic pathogen responsible for a wide range of infections in both humans and animals. In this study, we report the draft genome sequence of *K. pneumoniae* strain KP-S-66, isolated from soil samples collected from the vicinity of a cattle shed. Whole-genome sequencing was performed using the Illumina NextSeq 2000 sequencing platform. The assembled draft genome comprises 5175,725 bp distributed across 34 contigs, with an N_50_ value of 371,700 bp. Genome annotation detected 32 antibiotic resistance genes (ARGs), with a predominance of genes involved in multidrug resistance (MDR) efflux pump mechanisms. *In silico* pathogenicity analysis showed a pathogenicity score of 0.98 (out of 1), indicating a high pathogenic potential despite its environmental origin. The draft genome sequence of strain KP-S-66 has been deposited in the National Center for Biotechnology Information (NCBI) GenBank under the accession numbers JBRZGN000000000, PPRJNA1356658, SAMN53065372 and SRR35934007 for Genome, BioProject, BioSample and sequence read archive (SRA), respectively.

Specifications Table

**Table:** 

Subject	Bacterial sequencing
Specific subject area	Whole genome sequencing of MDR bacteria
Type of data	Raw reads of the sequenced genome
Data collection	Illumina NextSeq 2000 sequencing platform
Data source location	City/Town/Region: Dhamrai, Savar, Dhaka Country: Bangladesh Latitude and longitude for collected samples/data: 23.90° N, 90.22° E
Data accessibility	The data is hosted on NCBI BioProject: PRJNA1356658 (https://www.ncbi.nlm.nih.gov/bioproject/PRJNA1356658) BioSample: SAMN53065372 (https://www.ncbi.nlm.nih.gov/biosample/SAMN53065372) SRA: SRR35934007 (https://trace.ncbi.nlm.nih.gov/Traces/?run=SRR35934007)
Related research article	None

## 1 Value of the Data

• The whole-genome sequence of *K. pneumoniae* KP-S-66 provides valuable genomic insights into an environmental strain isolated from soil in the vicinity of a cattle farm.
• The dataset reveals the presence of 32 ARGs, enabling comparative analyses between environmental and clinical *K. pneumoniae* isolates.
• These data will help us studying ARGs transmission, environmental reservoirs, and the evolutionary dynamics of *K. pneumoniae* within a One Health framework.

## 2 Background

*K. pneumoniae* is an opportunistic Gram-negative bacterium with an increased capacity to acquire MDR, making it an emerging global threat to both public health and veterinary medicine [1,2]. Although MDR *K. pneumoniae* infections are predominantly associated with healthcare settings, convergent evidence identifies non-clinical reservoirs as critical nodes for the amplification and horizontal dissemination of resistance determinants [3,4]. Agricultural ecosystems, particularly soils interfacing with livestock operations, are subjected to a complex consortium of antimicrobial residues, fecal bacteria, and mobile genetic elements, creating a selective milieu conducive to the maintenance and recombination of resistomes [[5], [6], [7]]. Livestock farming operations, particularly in cattle and dairy production, represent critical reservoirs for MDR pathogens like *K. pneumoniae*, which persist in environmental matrices such as soil, manure, and bedding, thereby facilitating dissemination within agricultural ecosystems [[8], [9], [10]]. Whole-genome sequencing (WGS) has emerged as a powerful tool for the molecular characterization of MDR bacteria, enabling comprehensive analysis of genomic architecture, ARGs, and adaptive mechanisms that facilitate survival beyond clinical settings [[11], [12], [13]]. However, genomic data on environmental *K. pneumoniae* isolates, especially those originating from agricultural and livestock ecosystems in low- and middle-income countries remain limited. Therefore, genomic profiling of environmental MDR *K. pneumoniae* provides critical insights into environmental AMR reservoirs and supports One Health-based surveillance strategies and risk assessment frameworks.

## 3 Data Description

**Fig. 1:**
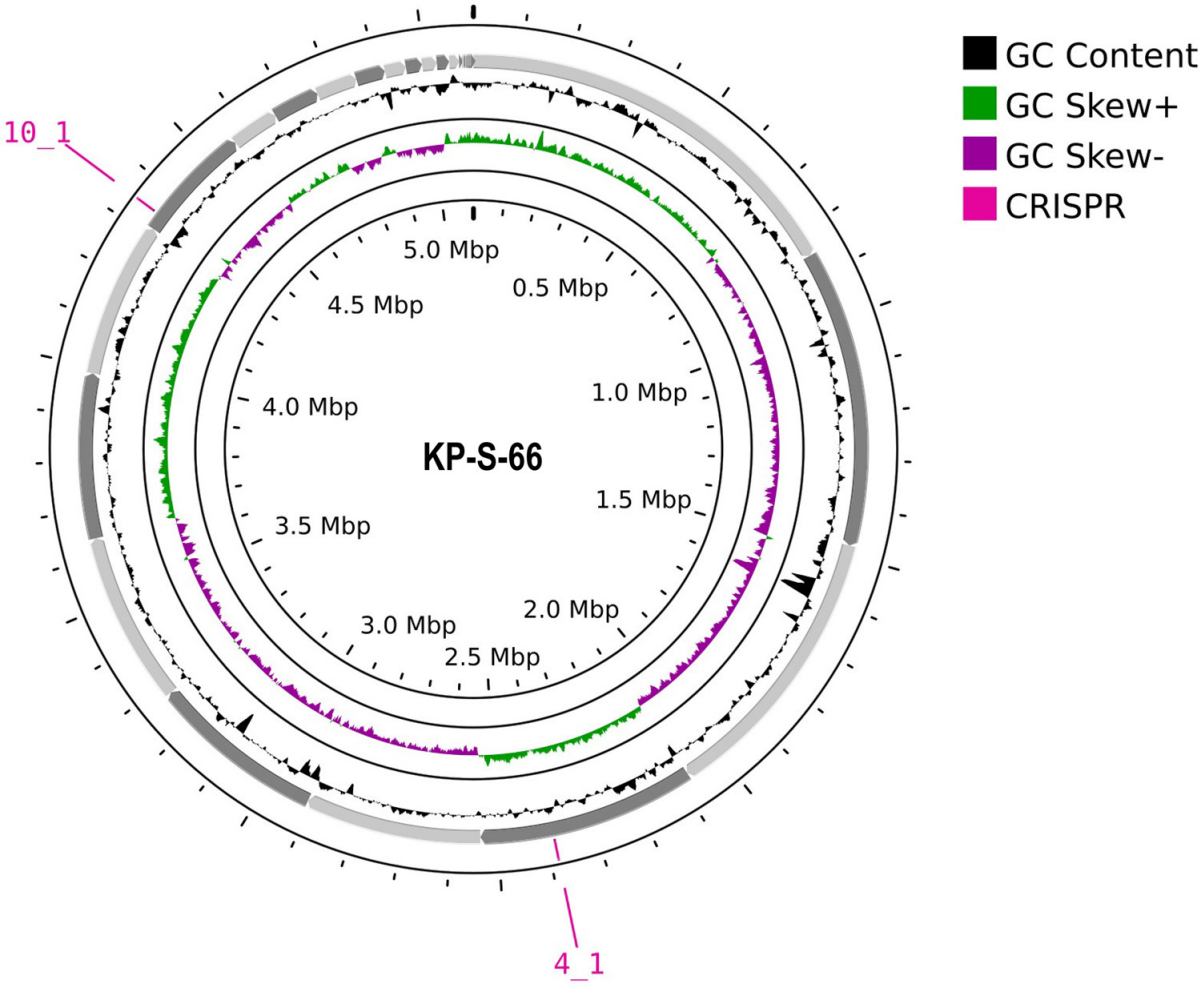
Circular map representation of genomic features of *K. pneumoniae* strain KP-S-66. From the outer to the innermost circles: GC content (black), GC skew positive (green) and negative (purple), and CRISPR loci (pink). The scale indicates genome size in megabase pairs (Mbp). The circular genomic map was created using the Proksee server (https://proksee.ca/).

This study represents the draft genome sequence and associated resistome features of *K. pneumoniae* strain KP-S-66, an environmental isolate obtained from soil collected near cattle shed in Dhamrai upazila, Savar (23.90° N, 90.22° E), Bangladesh. WGS followed by *in silico* analysis was performed to characterize genomic insights, AMR determinants, pathogenicity, functional subsystems, and CRISPR elements. The KP-S-66 genome exhibits a GC content of 57.5%, 99.04% genome completeness (0.93% contamination) and a sequencing coverage depth of 61.7×, both of which are consistent with values reported for publicly available *K. pneumoniae* genomes [1,14,15]. Genome annotation revealed 5036 genes, including 4933 protein-coding sequences (CDSs) and 80 tRNA genes. Thirteen rRNA genes were detected, comprising nine copies of 5S rRNA, two copies of 16S rRNA, and two copies of 23S rRNA. In addition, the genome harbors 10 non-coding RNAs (ncRNAs) and 104 pseudogenes. Collectively, these genomic features are in agreement with previously reported *K. pneumoniae* genomes, and support the completeness and reliability of the draft assembly. The circular genome map of strain KP-S-66 (Fig. 1) shows a relatively uniform GC content across the chromosome, with distinct transition points in GC skew. Two CRISPR loci were identified at separate chromosomal locations; however, no associated *cas* genes were detected, suggesting the existence of orphan CRISPR locus patterns. The *K. pneumoniae* strain KP-S-66 was confidently assigned to serotype O1 (O1/O2v2 locus) and capsular type KL58. Both loci were fully intact: the O-locus had 100% gene presence (99.44% nucleotide identity) and the wbbY gene confirmed O1 over O2. All core O-antigen genes were present. The KL58 locus was complete (22/22 genes, 99.40% identity), including assembly, glycosyltransferase, and sugar biosynthesis genes. No truncations were found. Thus, KP-S-66 is genetically equipped to produce functional surface polysaccharides essential for structure, immune evasion, and pathogenicity. No plasmid replicons or known plasmid-associated antimicrobial resistance genes were detected in the genome of *K. pneumoniae* strain KP-S-66. However, the genome exhibited a high pathogenicity score (0.9817), suggesting a strong likelihood of human pathogenic potential.

**Fig. 2:**
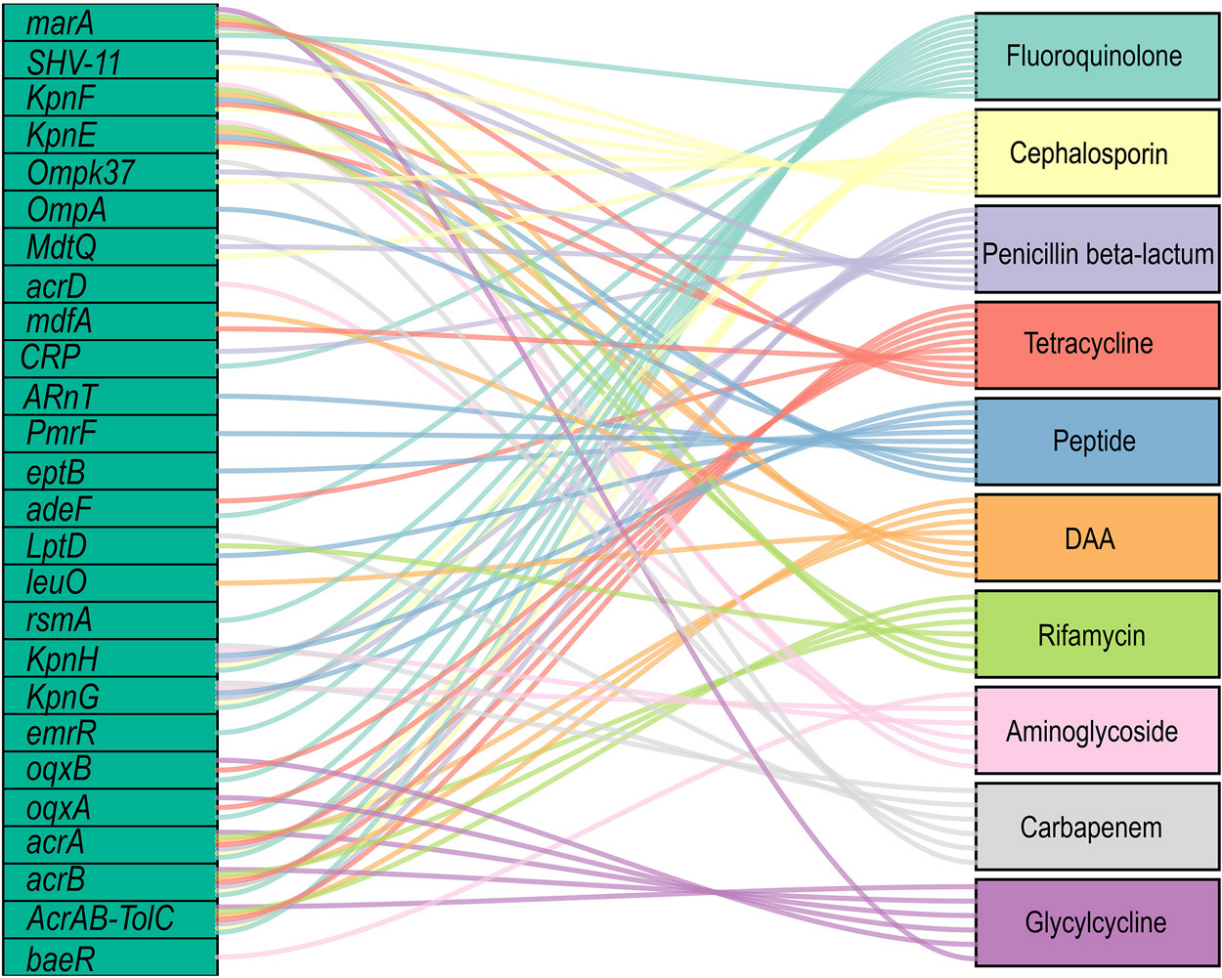
Resistome profile of *K. pneumoniae* strain KP-S-66. The Sankey diagram depicts the associations between identified antimicrobial resistance genes (ARGs) (left) and their corresponding antibiotic classes (right). Flow widths indicate functional links between resistance determinants and the top 10 antibiotic categories to which they confer resistance. Here, DAA refers to disinfecting agents and antiseptics.

**Fig. 3:**
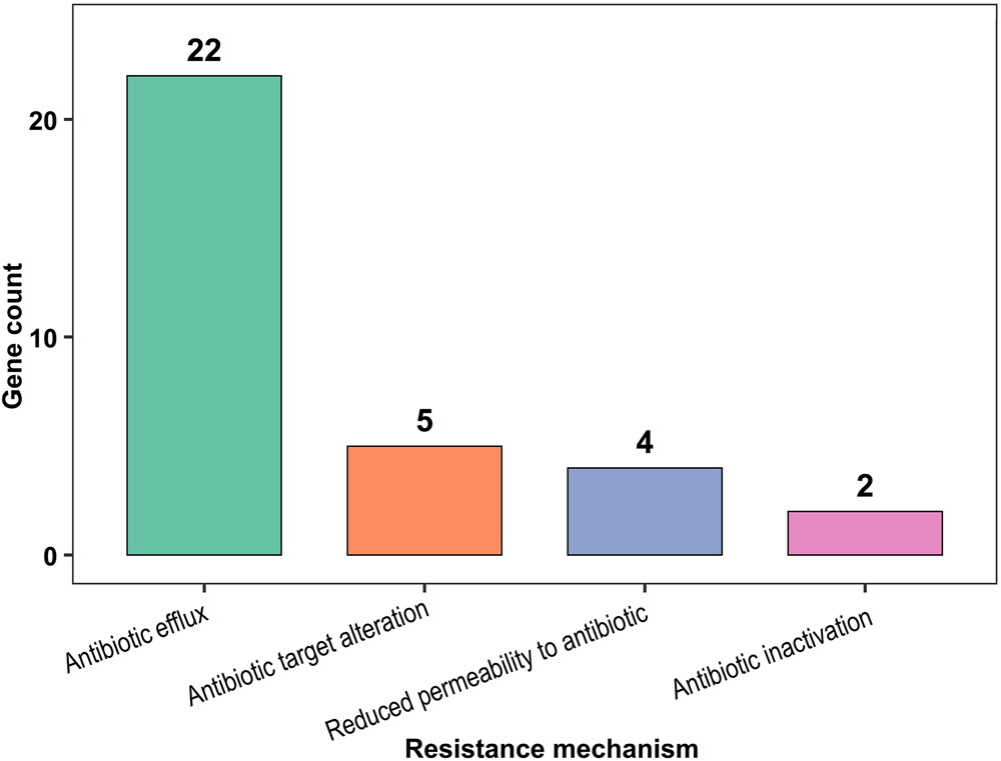
Distribution of antimicrobial resistance mechanisms identified in *K. pneumoniae* strain KP-S-66. The X-axis represents resistance mechanisms, and the Y-axis shows the number of antibiotic resistance genes in each category. Antibiotic efflux (green) was the predominant resistance mechanism, followed by antibiotic target alteration (orange), reduced permeability to antibiotics (blue), and antibiotic inactivation (pink).

Resistome analysis revealed a diverse repertoire of 32 ARGs conferring resistance to 20 distinct antibiotic classes. The resistome was largely dominated by multidrug resistance (MDR) efflux pump–associated genes, including *AcrAB-TolC, OqxAB, Mdt*, and *Emr*, along with critical regulatory elements such as *marA, baeR, ramA*, and *crp*, underscoring a strong efflux-driven resistance potential. The Sankey diagram (Fig. 2) illustrates the relationships between individual ARGs and their corresponding antibiotic classes, highlighting the MDR potential of strain KP-S-66, mainly driven by broad-spectrum efflux pumps and their regulatory systems. In addition to efflux pump-based mechanisms, genes associated with target modification, reduced membrane permeability, and antibiotic inactivation were also detected. The bar plot depicting the distribution of resistance mechanisms (Fig. 3) indicates that antibiotic efflux represents the dominant resistance strategy (66.67%), followed by target alteration (15.15%), decreased permeability (12.12%), and antibiotic inactivation (6.07%). Collectively, these findings underscore the central role of efflux-mediated resistance as a key adaptive strategy enabling environmental *K. pneumoniae* strains to withstand diverse antimicrobial pressures. Despite having generated a high-quality draft genome with robust completeness and coverage, this study is limited by the analysis of only a single environmental isolate (KP-S-66) and the exclusive use of in silico predictions without phenotypic validation, requiring future validation through phenotypic assays and larger, more diverse isolate collections from multiple sources to confirm the resistome and functional characteristics reported in this article. Overall, this study provides comprehensive insights into the genomic architecture, AMR determinants, and CRISPR arrays of *K. pneumoniae* KP-S-66, offering a valuable resource for comparative genomics, AMR ecology, surveillance initiatives, and One Health–oriented research on antimicrobial resistance.

## 4 Experimental Design, Materials and Methods

### 4.1 Sample collection and bacterial isolation

Soil samples were collected in August 2023 from an area adjacent to cattle shed in Dhamrai upazila, Savar, Bangladesh, following aseptic and standard microbiological procedures. Samples were aseptically transferred into sterile containers containing buffered peptone water (Oxoid, UK), and transported to the laboratory under cold-chain conditions. Upon arrival, samples were stored at 4 °C and processed within 12 h to preserve microbial viability. Isolation of *K. pneumoniae* was performed in accordance with ISO/TS 21,872–1:2023. Briefly, enriched soil suspensions were plated onto Simmons Citrate Agar (Oxoid, UK) supplemented with 1% inositol and incubated aerobically at 37 °C for 24 h. Presumptive *K. pneumoniae* colonies were identified based on characteristic yellow, dome-shaped, mucoid morphology, and purified by subculturing. Species-level confirmation was carried out using the VITEK® 2 Compact system (bioMérieux, USA), and PCR amplification with *K. pneumoniae*-specific primers. A confirmed isolate was subsequently selected for genome sequencing and downstream genomic analyses.

### 4.2 DNA extraction, library preparation and sequencing

Genomic DNA was extracted from a purified culture of *K. pneumoniae* using the Qiagen DNeasy Blood & Tissue Kit (Qiagen, USA) following a modified CDC PulseNet total DNA extraction protocol. Prior to DNA purification, bacterial cells were harvested by centrifugation at 14,000 rpm for 5 min. The purity of the extracted DNA was evaluated using a NanoDrop Spectrophotometer 2000 (Thermo Fisher Scientific, USA), where 260/280 nm ratios of approximately 1.8 and 260/230 nm ratios of approximately 2.0 indicated high-quality DNA with minimal contamination. DNA concentration was quantified using a Qubit 4.0 Fluorometer (Thermo Fisher Scientific, USA) according to the manufacturer’s instructions. Sequencing libraries were prepared using the Illumina DNA Prep Reagent Kit with an automated liquid handling system (epMotion 5075). The prepared libraries were sequenced on the Illumina NextSeq 2000 platform using paired-end 2 × 150 bp chemistry to generate high-quality reads for downstream whole-genome assembly and analysis.

### 4.3 Genome assembly and annotation

Raw read quality was evaluated with FastQC v0.11.3 [16], and reads with low quality were subsequently trimmed with Trimmomatic v0.39.2 [17]. High quality reads (Phred score > 30) were assembled using SPAdes v4.0.0 [18], and annotated through the NCBI Prokaryotic Genome Annotation Pipeline v6.6 [19]. Genome completeness was assessed using CheckM v1.2.3 [20]. Kleborate v3.2.4 was used to predict the O- and K- loci in the assembled genome [21]. CRISPR arrays were detected using CRISPRCasFinder v4.2.20 [22]. The distribution of subsystems was determined via the RAST v2.0 server [23], and the potential pathogenic risk to humans was assessed using PathogenFinder v1.1 [24]. PlasmidFinder v2.0.1 [25] was used to identify plasmid replicons and known plasmid-associated resistance genes. Default parameters were applied for all tools unless specified otherwise.

### 4.4 Resistome analysis and visualization

Resistomes (ARGs and concurrent mechanisms) in the assembled genome were analyzed by screening the genome against the Comprehensive Antibiotic Resistance Database (CARD) v3.2.4 [26], integrated into the ABRicate pipeline (https://github.com/tseemann/abricate). Circular genome map was generated using CGView web tool [27], and additional data visualizations were created in R v4.5.0 [28].

## Limitations

A key limitation of this study is the small sample size, as only a single *K. pneumoniae* genome was analyzed. This limited representation may affect the generalizability of the findings and the robustness of conclusions, particularly with respect to resistome profiling, pathogenic potential, and comparative genomic interpretations.

## Ethics Statement

The authors have read and followed the ethical requirements for publication in Data in Brief and confirm that the current work does not involve human subjects, animal experiments, or any data collected from social media platforms.

## Author Contributions

**Sonia Akther:** Project Administration, Conceptualization, Investigation, Data curation, Visualization, Writing- Original draft preparation. **Md Abu Ahsan Gilman:** Conceptualization, Methodology, Formal analysis, Writing-review & editing, Visualization, Investigation, Data curation. **Md. Niamul Shahadat:** Methodology, Investigation, Data curation, Formal analysis, Validation. **Mst Sogra Banu Juli:** Methodology, Investigation, Data curation, Formal analysis, Validation. **M. Nazmul Hoque:** Project Administration, Funding Acquisition, Conceptualization, Methodology, Writing-review & editing.

## Data Availability

NCBI) GenBankKlebsiella pneumoniae strain KP-S-66 Genome Sequencing (Original data).
